# A Systematic Review of the Effects of Single-Event Multilevel Surgery on Gait Parameters in Children with Spastic Cerebral Palsy

**DOI:** 10.1371/journal.pone.0164686

**Published:** 2016-10-18

**Authors:** Robert P. Lamberts, Marlette Burger, Jacques du Toit, Nelleke G. Langerak

**Affiliations:** 1 Division of Orthopaedic Surgery, Department of Surgical Sciences, Faculty of Medicine and Health Sciences, Stellenbosch University, Tygerberg, South Africa; 2 Division of Exercise Science and Sports Medicine, Department of Human Biology, Faculty of Health Sciences, University of Cape Town, Newlands, South Africa; 3 Division of Physiotherapy, Department of Interdisciplinary Health Sciences, Faculty of Medicine and Health Sciences, Stellenbosch University, Tygerberg, South Africa; 4 Division of Neurosurgery, Department of Surgery, Faculty of Health Sciences, University of Cape Town, Cape Town, South Africa; IRCCS E. Medea, ITALY

## Abstract

**Background:**

Three-dimensional gait analysis (3DGA) is commonly used to assess the effect of orthopedic single-event multilevel surgery (SEMLS) in children with spastic cerebral palsy (CP).

**Purpose:**

The purpose of this systematic review is to provide an overview of different orthopedic SEMLS interventions and their effects on 3DGA parameters in children with spastic CP.

**Methods:**

A comprehensive literature search within six databases revealed 648 records, from which 89 articles were selected for the full-text review and 24 articles (50 studies) included for systematic review. The Oxford Centre for Evidence-Based Medicine Scale and the Methodological Index for Non-Randomized Studies (MINORS) were used to appraise and determine the quality of the studies.

**Results:**

Except for one level II study, all studies were graded as level III according to the Oxford Centre for Evidence-Based Medicine Scale. The MINORS score for comparative studies (n = 6) was on average 15.7/24, while non-comparative studies (n = 18) scored on average 9.8/16. Nineteen kinematic and temporal-distance gait parameters were selected, and a majority of studies reported improvements after SEMLS interventions. The largest improvements were seen in knee range of motion, knee flexion at initial contact and minimal knee flexion in stance phase, ankle dorsiflexion at initial contact, maximum dorsiflexion in stance and in swing phase, hip rotation and foot progression angles. However, changes in 3DGA parameters varied based on the focus of the SEMLS intervention.

**Discussion:**

The current article provides a novel overview of a variety of SEMLS interventions within different SEMLS focus areas and the post-operative changes in 3DGA parameters. This overview will assist clinicians and researchers as a potential theoretical framework to further improve SEMLS techniques within different SEMLS focus groups. In addition, it can also be used as a tool to enhance communication with parents, although the results of the studies can’t be generalised and a holistic approach is needed when considering SEMLS in a child with spastic CP.

## Introduction

Gait abnormalities are common in children with cerebral palsy (CP) and are generally caused by an abnormal muscle tone, loss of motor control and balance problems due to a non-progressive lesion of the developing brain [1]. Following the natural progression of skeletal and muscle growth in CP, these children often develop secondary abnormalities, resulting in further deterioration of their walking pattern [1,2].

The assessment and treatment of gait abnormalities in children with CP are challenging. Several complementary interventions are often used to develop the most optimal and energy efficient gait pattern in these children. These interventions range from physical and occupational therapy, neurosurgical and pharmacological interventions to reduce hypertonia and orthopedic interventions aiming to restore anatomical structures and musculoskeletal conditions [3].

As a multi-level approach has proven to be the most effective treatment option, it is not surprising that within orthopedics, single-event multilevel orthopedic surgery (SEMLS) is the preferred method to treat musculoskeletal deformities in children with CP [3,4]. SEMLS is defined as corrections of soft tissue and/or bony deformities at a minimum of two anatomical levels, during a single operative event. The advantage of a SEMLS procedure, in contrast to multiple series of interventions, is that only one hospital admission and recovery period are needed for multiple interventions

Recently, McGinley et al. [4] conducted a systematic review that aimed to determine which outcomes measures are frequently used to assess the effectiveness of SEMLS in children with CP. The finding of this study showed that 3-dimensional gait analysis (3DGA), and more specifically kinematic and temporal-distance parameters, are most commonly used to assess the effectiveness of SEMLS interventions. However, this review did not provide an overview of changes in 3DGA parameters after SEMLS interventions in children with spastic CP.

Providing an overview of which changes in gait parameters can be expected after SEMLS, is of great value for clinicians and researchers. Unfortunately, and due to the variety of SEMLS interventions with different focus areas and specific CP populations, it is impossible to perform a meta-analyses of the literature. However, a systematic review of SEMLS studies and their effect on 3DGA parameters in children with CP will result in an overview of gait changes that have been reported after different types of SEMLS techniques. A systematic review of these SEMLS outcome studies can provide valuable insight for clinicians, can assist in preoperative discussion with parents and form a platform to potentially further improve SEMLS techniques Therefore the aim of this study is to provide a systematic overview of which soft tissue and bony interventions have been performed as part of SEMLS intervention in children with CP, with a special focus on the post-operative changes in 3DGA kinematic and temporal-distance parameters.

## Methods

### Database sources and search

A systematic review was conducted in accordance with the Preferred Reporting Items for Systematic Reviews and Meta-Analyses (PRISMA) guidelines [5]. A comprehensive literature search of six computerized bibliographic databases accessed through the Stellenbosch University library services was conducted. These databases include Medline, Cochrane Library, Canal, Proquest, Science Direct and Scopus. Specific search strategies were tailored for each database, using MeSH terms and/or single concepts, their synonyms as well as combining Boolean operators where available. The following key search terms were used: (“cerebral palsy” AND “gait”) AND (“orthopaedic surgery” OR “orthopedic surgery” OR “orthopaedics” OR “orthopedics” OR “surgery”). The searches were limited to humans only, articles written in English and published between January 1985 and December 2015.

### Selection of papers

Articles were evaluated for eligibility based on the title and abstract. After the initial identification and screening, full-text articles were reviewed and independently assessed against the inclusion/exclusion criteria by two reviewers (NL and MB). Articles were selected when fulfilling the following criteria: 1) Study the effects of SEMLS on gait assessed by 3DGA; 2) Cohort consisted of ambulant children and adolescents diagnosed with spastic CP; 3) Only SEMLS interventions performed (e.g. not combined with botulinum toxin injections 6 months prior to surgery); 4) Detailed description of orthopedic procedures; 5) Reported number of operated sides; 6) 3DGA conducted before and after SEMLS interventions; 7) Mean follow-up time of at least 12 months; and 8) Include at least three temporal-distance and/or kinematic parameters.

### Quality assessment

Two reviewers (NL and MB) graded the level of evidence of the selected articles by using the Oxford Centre for Evidence-Based Medicine Scale [6] and completed the quality appraisal with Methodological Index for Non-Randomized Studies (MINORS) [7]. The MINORS tool consists of a checklist ofeight items specifically designed for non-comparative studies and four additional items for use within comparative studies. Items on the MINORS tool are scored as 0 (not reported), 1 (reported but inadequate) and 2 (reported and adequate), resulting in a total score of 16 for non-comparative studies and 24 for comparative studies. Each study was independently reviewed by two authors (NL and MB), after which the scores were compared and decided on final scores during a consensus meeting.

### Data synthesis and analysis

Two reviewers (NL and RL) extracted the demographic and SEMLS background information, as well as the pre- and post-operative temporal-distance and kinematic parameter data. Temporal-distance and kinematic gait parameters were included if they were used in at least 7 different studies. In addition, reference norm values from typical developing children were extracted where possible. Significant changes were defined as ‘improved’, a significant change getting closer to the reference norm values, or as ‘deteriorated’, a significant change moving away from the reference norm values. An experienced pediatric orthopedic surgeon (JDT) reviewed the selected articles with regards to data on surgical interventions performed, while a fourth reviewer (MB) verified all extracted information.

## Results

### Database sources, search and quality assessment

**Fig 1:**
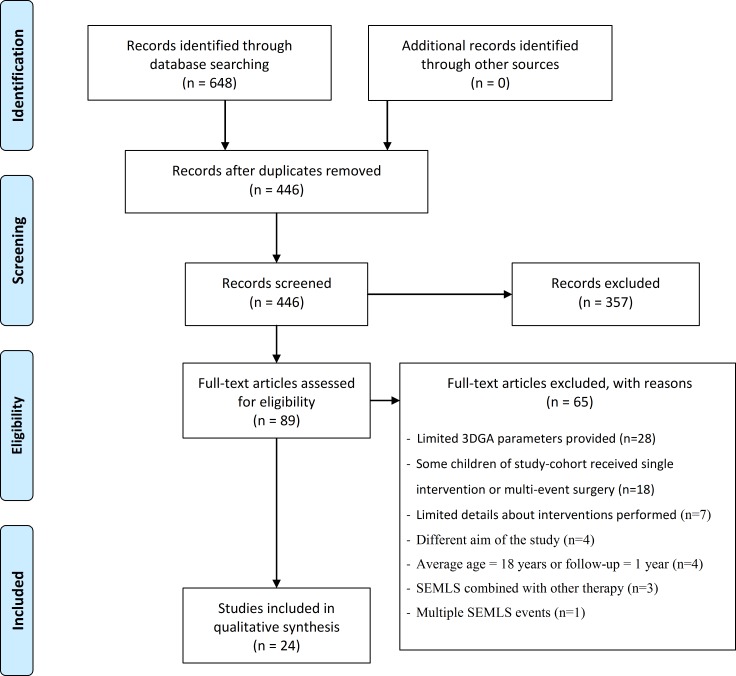
Flowchart of the search strategy.

The electronic databases search produced 648 initial references of which 24 articles met the inclusion criteria (Fig 1) [8–31]. Thirteen of the 24 articles reported on multiple follow-up assessments or different study cohorts [8,11,13,14,16,17,21,23,24,26,27,30,31], resulting in 50 different studies included in this systematic review.

All the articles were graded as OXFORD [6] level III studies, except for the study by Dreher et al. [23], who conducted a randomized control trial (RCT), which was graded as a level II. The MINORS scores [7] for the methodological quality appraisal of the articles are presented in Table 1. Eighteen of the 24 included articles were non-comparative prospective or retrospective cohort studies with an average MINORS score of 9.8 (range 5–13). The remaining 6 articles compared different interventions between groups, with an average MINORS score of 15.7 (range 15–17). Although all comparative studies used strict selection criteria, only two of these studies used factors to match their comparative groups. Thompson et al. [31] matched the groups based on GMFCS levels (Level I-III), while Dreher et al. [30] used primary (knee flexion and ankle dorsiflexion in stance) and secondary (pelvic tilt, hip flexion, age at surgery, body mass index (BMI), Gillette Gait Index (GGI) and GMFCS level) factors to match their two interventions groups.

**Table 1:** Overview of selected articles with appraisal scores, study cohort characteristics and surgical interventions details expressed as the percentage (%) of total operated sides.

Authors	CP type	Age	Sides	Soft-tissue interventions											Bony interventions				MINOR
	n	(years)	n^group^	Psoas	Ham Med	Ham Lat	Add	RF Tr	RF Rel	Apneu GasSol	Apneu Gas	TAL	Foot Soft	Other	FDO	TDO	Foot Bony	Other	(score / total)
**General multi-level surgeries**																			
Dreher et al.[8]	30D	10.3	60^HM^	12%	100%		12%	93%	50%	75%^a^			23%		63%	3%	52%		15 / 23
	9D		18^HL^	39%	100%	100%	17%	83%	33%	50%^a^			22%		56%	17%	56%		
Rutz et al.[9]	13D	12.8	26	15%	92%	15%	46%		12%	35%		19%	23% ^e^	46% ^l^	35%		23%		9 / 15
Saraph et al.[10]	25D	13.6	50	68%	92%	20%	14%	92%		34%			44%		24%	12%	8%		13 / 16
Saraph et al.[11]	32D	11.1	64	66%	86%	22%	14%	86%		36%			44%		28%	13%	11%		10 / 15
Zwick et al.[12]	17D	11.2	34	62%	31%	18%	18%	91%		32%			24%		24%	9%	6%		11 / 16
**Lever arm dysfunction**																			
Dreher et al.[13]	33D	10.5	66	18%	97%	21%		91%	27%	72%^a^					89%	12%	27%		8 / 15
Saraph et al.[14]^$^	8D	11.9	16^D^	100%	100%	62%	25%	50%		100%			75%		50%	25%	25%		13 / 16
	14H		14^H^	100%	100%	30%	40%	100%		30%			110%		100%	40%	30%		
Dobson et al.[15]	17H	12.1	17	12%	76%	12%	47%	71%			35%	41%	18% ^e^		100%				11 / 16
Ounpuu et al.[16]	18D/Q, 2H	8.1	27	30%	100%	19%	37%	56%	41%		85%		30% ^f^		100%	4%			8 / 16
Kay et al. [17]	16D/Q, 3H	9.7	19^FDO^	32%	58%		32%	62%			37%	5%			100%				15 / 23
	25D/Q, 15H		40^NFDO^	45%	53%		28%	60%			40%	18%	5%^e^_,_ 8%^g^, 3%^h^					3%	
**Multi-level tendon lengthening surgery**																			
Steinwender et al.[18]	16D	10.2	29	18%	100%	17%		100%					14%						11 / 16
Adolfsen et al.[19]	20D, 1Q, 10H	8.5	39	13%	100%	8%		100%			85%	15%	15%^e^, 26%^k^^,^^h^^,^^i^						8 / 15
Bernthal et al.[20]	23Amb	9.2	40	65%	100%		35%					40%							13 / 16
**Gait pattern**																			
Rodda et al.[21]	10D	12.0	20	40%	100%	60%	20%	40%							45%	20%	25%		9 / 15
Cruz et al.[22]	33D, 5Q, 4H	8.5	69	7%	78%	9%	12%		100%	45%^b^		45%^b^	3%^e^		32%	25%			9 / 15
Dreher et al.[23]	15D	10.3	30^RF^	20%	30%			100%		83%^a^			17%	33%^l^	73%	7%	33%		17 / 23
	17D	11.9	34^NRF^	32%	32%					73%^a^			9%	41%^l^	79%	6%	41%		
Dreher et al.[24]	33D	10.1	66^RF^	12%	85%	6%		100%	30%	76%^a^			35%, 20% ^f^		53%	9%	32%		15 / 23
	20D	11.8	40^PRF^	35%	91%	40%		100%	70%	70%^a^			35%, 20% ^f^		63%	10%	55%		
Presedo et al.[25]	45D	13.3	80	34%	100%		84%		100%	60%			8%^e^_,_ 13%^i^_,_ 28%^j^		75%	26%	16%		9 / 15
**Operative techniques**																			
Svehlík et al.[26]	18D	11.5	21	86%	86%	38%	24%	86%					90%		52%	33%	29%		9 / 15
Dreher et al.[27]	44D	9.8	82	13%	83%			96%	39%	41%	59%	2%			71%	7%	28%		9 / 16
Saraph et al.[28]	22D	12.6	28	71%	114%	29%	21%	114%		100%			93%^k^	7%^m^	50%	29%	93%^k^		5 / 15
Metaxiotis et al.[29]	20D	11.5	40	23%	65%	25%	15%	100%	68%		100%^c^,60%^d^		25%, 100%^f^	105%	25%	5%	15%	5%^n^	12 / 16
Dreher et al.[30]	21D	11.3	42^CBM^	17%		14%	14%	100%	100%	29%		10%	21%, 200%^f^		41%	12%	21%		15 / 23
	21D	11.1	42^MTL^	14%		21%	24%	91%	43%	69%		10%	21%, 200%^f^		67%	12%	36%		
Thompson et al.[31]	10D	10.6	18^MI^		94%		89%	89%			72%		6%^e^		100%	6%	39%		15 / 23
	10D	11.4	20^NMI^	50%	95%		15%	50%			95%		15%^e^		85%		40%		

*Abbreviations*: **CP Type)** D, diplegic; H, hemiplegic; Q, quadriplegic; Amb, ambulatory; **Sub-groups)** HM, medial hamstrings lengthening; HL, combined medial and lateral hamstrings lengthening; FDO, femoral derotation osteotomy; NFDO, no femoral derotation osteotomy; RF, distal rectus femoris transfer; PRF, prophylactic distal rectus femoris transfer; NRF, no distal rectus femoris transfer; CBM, conversion of biarticular muscles; MTL, multi-tendon lengthening; MI, minimally invasive SEML techniques; NMI, no minimally invasive SEML techniques. **Interventions)** Psoas, psoas lengthening; HamMed, medial hamstrings lengthening; HamLat, lateral hamstrings lengthening; Add, adductor lengthening; RF Tr, rectus femoris transfer; RF Rel, rectus femoris release; Apneu GasSol, apneurotic gastrocneumius-soleus muscle lengthening; Apneu Gas, apneurotic gastrocnemius lengthening; TAL, tendon achilles lengthening; FootSoft, foot tendon lengthening and transfers; FDO, femoral derotation osteotomy; TDO, tibia derotion osteotomy; FootBony, foot osteotmies. *Icons*: ^*$*^ *Number (%) of interventions is estimated from average per subject*. **Other interventions)**

^a^Calf muscle lengthening

^b^Apneu GasSol or TAL

^c^Proximal gastrocnemius transfer

^d^Intramuscular gastrocnemius transfer

^e^Tibialis posterior interventions

^f^Semitendinosus intervention

^g^Toe tendons lengthening

^h^Plantar fascia releases

^i^Tibialis anterior transfer

^j^Peroneus brevis lengthening

^k^Soft and or bony foot surgeries

^l^Patella tendon shortening

^m^Knee capsulotomy

^n^Pelvic osteotomy.

### Studies, focus areas, SEMLS characteristics and participants

Table 1 provides an overview of the 50 studies conducted within the 24 selected articles. All articles were published by a range of international research groups based in different countries (Austria (n = 7) [10–12,14,18,26,28], USA (n = 5) [16,17,19,20,22], Australia (n = 2) [15,21], Germany (n = 7) [8,13,23,24,27,29,30], Switzerland (n = 1) [9], France (n = 1) [25] and the United Kingdom (n = 1) [31]). The focus area of the SEMLS intervention ranged from General multi-level surgeries, Lever arm dysfunction, Multi-level tendon surgeries, Gait pattern to Operative techniques, which resulted in a variety of type of interventions as part of the SEMLS as presented in Table 1.

The sample size of the different study cohorts ranged from 8 to 45 children (14–82 operated sides) with a mean age between 8 and 13 years. Except for one article [20], all authors classified the type of CP of their participants. Seventeen articles studied the effects of SEMLS in children with spastic diplegia [8–13,18,21,23–31], one article focused on children with hemiplegia [15], while one article compared diplegia with hemiplegia [14]. In addition, four articles studied children with unilateral and/or bilateral type of CP [16,17,19,22]. An overview of the specific inclusion and exclusion criteria, as well as information about the 3DGA are presented in Table 2. The mean follow-up time after SEMLS intervention ranged from 1.0 to 9.1 years as shown in Tables 3–7.

**Table 2:** Overview of the inclusion and exclusion criteria and, 3D gait analyses (3DGA) capturing details.

Authors	Inclusion criteria	Exclusion criteria	3DGA details				
			Speed	Distance	Trials (n)	Condition	*Other*
**General multi-level**							
Dreher et al.[8]	D; ambulant; flexed knee gait	History of orthopedic surgery; dyskinetic CP; B-T-A last 6 months; severe mental retardation.	Self-selected				
Rutz et al.[9]	D; age: 6–18 years; GMFCS Level I, II, III	B-T-A in last 6 months; dystonic or mixed movement disorder.	Self-selected		≥6		
Saraph et al.[10]	D; ambulant; good vision; no walking aid; comprehend instructions	History of orthopedic surgery.	Self-selected	12m	≥5		Force plate contact
Saraph et al.[11]	D; ambulant; good vision; no walking aid; comprehend instructions	History of ortopedic surgery.	Self-selected	12m	≥5		Force plate contact
Zwick et al.[12]	D; ambulant; good vision; no walking aid (10 min); comprehend instructions	History of orthopedic surgery; mental retardation; athetoid.		12 m	≥5		Force plate contact
**Lever arm dysfunction**							
Dreher et al.[13]	D: GMFCS Level I, II, III; internally rotated gait	History of orthopedic surgery; B-T-A last 6 monhs; other consecutive surgery.	Self-selected	7m			Force plate contact
Saraph et al.[14] ^$^	D, H; ambulant; good vision; no walking aid; comprehend instructions; fixed bony internal rotation (hip)	Hip dysplasia or excessive coxa valga that requires proximal femoral or additional pelvic osteotomies.	Self-selected	12m	≥5		Force plate contact
Dobson et al.[15]	H	Bony surgeries except for equinus deformity.	Self-selected	10m	≥3	Barefoot	Force plate contact
Ounpuu et al.[16]	D, H, Q; ambulant; FDO	History of orthopedic surgery.	Self-selected	10m	≥3	Barefoot	Force plate contact
Kay et al.[17]	D, H, Q with static encephalopathy; ambulant; soft tissue surgery with and without FDO	Concomitant tibial osteotomies; foot surgery.	Self-selected	15m	≥3		
**Multi-level tendon length.**							
Steinwender et al.[18]	D; ambulant; no walking aid (10 minutes); comprehend instructions; spastic internal rotation gait	History of orthopedic surgery; moderate to severe mental retardation; athetoid.					
Adolfsen et al.[19]	D, H, Q; ambulant; age: 5–15 years; simultaneous medial hamstring lengthening, rectus femoris transfer, gastrosoleus lengthening surgeries	Femoral and tibial derotational osteotomies.	Self-selected	9m	≥3	Barefoot	Force plate contact
Bernthal et al.[20]	ambulant (household or community level); age: 4–18 years; one or more indications for soft tissue surger.	Single level surgery.	Self-selected	15m			Without brace
**Gait pattern**							
Rodda et al.[21]	D; age: 4–18 years; GMFCS level II, III; severe crouch gait; with/without walking aid.	SDR; intrathecal Baclofen pump; B-T-A in last 12 months.	Self-selected	10m	≥3	Barefoot	Force plate contact
Cruz et al.[22]	CP; ambulant; rectus femoris intramuscular lengthening.	Not described.	Self-selected		≥3		
Dreher et al.[23]	D; age: 6–16 years; GMFCS level I, II, III; distal rectus femoris transfer; decrease in romKFSw of at least 15˚; Duncan-Ely test: +; Tardieu scale ≥ 1	Previous lower limb surgery; SDR; dystonia.	Self-selected	7m		Barefoot	
Dreher et al.[24]	D; age: 6–16 years; GMFCS level I, II, III; distal rectus femoris transfer as part of SEMLS; positive Duncan-Ely test	History of orthopedic surgery; B-T-A in the last 6 months; dyskinetic.	Self-selected	7m	≥5	Barefoot	
Presedo et al.[25]	D related to prematurity; age: 4–18 years; with/without walking aid	Not described.	Self-selected		multiple		
**Operative techniques**							
Svehlík et al.[26]	D; GMFCS level: I, II, III; equines gait; Baumann procedure; walk barefoot and independently for 10 months	Non-spastic CP; History of orth. Surgery, SDR or intra-thecal baclofen.	Self-selected	10m	≥5	Barefoot	Force plate contact
Dreher et al.[27]	D; age: ≥6 at surgery; GMFCS level: I, II, III; fixed equines	History of orthopedic surgery; SDR; dyskinetic; B-T-A in last 6 months.	Self-selected	7m		Barefoot	
Saraph et al.[28]	D; good vision; no walking aids; fixed contracture of the gastrocnemius and the soleus; negative Silfverskiöld test	Not described.	Self-selected				
Metaxiotis et al.[29]	D; age: 5–17 years; no walking aids; comprehend instructions	History of orthopedic surgery in last 12 months; athetoid.	Self-selected	7m	8		
Dreher et al.[30]	D; ambulant; age: 6–16 years; GMFCS level: I, II, III; flexed knee gait	History of orthopedic lower limb surgery; B-T-A in last 6 months.	Self-selected	7m	≥5	Barefoot	
Thompson et al.[31]	D; age: 5–17 years; GMFCS level: I, II, III; no walking aids; comprehend instructions	History of orthopedic Surgery; B-T-A in last 12 months.	Self-selected	10m	≥6	Barefoot	

*Abbreviations*: D, Diplegic; H, Hemiplegic; Q, quadriplegic; GMFCS, Gross Motor Function Classification System; B-T-A, botulinum toxin A injections; romKFSw, range of motion of knee flexion during swing phase; m, meters.

**Table 3:** Changes in temporal distance parameters after SEMLS. Data expressed mean (standard deviation) and mean change.

Article	Follow-up time (yrs)	Sub-group	Stride length (cm)			Cadence (steps/min)			Velocity (cm/sec)		
			*Norm values*: *111–134 cm*			*Norm values*: *118–130 steps/min*			*Norm values*: *119–138 cm/sec*		
			Pre	Post	Mean change	Pre	Post	Mean change	Pre	Post	Mean change
**General multi-level surgery**											
Dreher et al.[8]	1.0	HM	82 (21)	79 (23)	-3	123 (26)	104 (27)	**-19**	83 (23)	70 (30)	**-13**
		HL	82 (12)	91 (23)	+9	113 (13)	88 (37)	-25	78 (21)	71 (41)	-7
	3.1	HM	82 (21)	90 (23)	+8	123 (26)	115 (25)	-8	83 (23)	88 (28)	+5
		HL	82 (12)	95 (26)	+13	113 (13)	104 (27)	-9	78 (21)	84 (37)	+6
	8.1	HM	82 (21)	99 (20)	**+17**	123 (26)	114 (20)	-9	83 (23)	96 (28)	+13
		HL	82 (12)	89 (24)	+7	113 (13)	104 (23)	-9	78 (21)	79 (35)	+1
Rutz et al.[9]	1.8		88 (19)	98 (20)	+10	186 (34)	183 (39)	-3	83 (26)	90 (27)	+7
Saraph et al.[10]	3.3		95 (14)	113 (11)	**+18**	134 (14)	126 (10)	**-8**	106 (23)	119 (13)	**+13**
Saraph et al.[11]	1.0		95 (18)	103 (16)	**+8**	134 (16)	132 (18)	-2	105 (23)	114 (20)	**+9**
	2.3		95 (18)	108 (12)	**+13**	134 (16)	127 (11)	**-7**	105 (23)	114 (14)	**+9**
	4.4		95 (18)	110 (11)	**+15**	134 (16)	124 (11)	**-10**	105 (23)	114 (15)	**+9**
Zwick et al.[12]	3.8		97 (15)	111 (12)	**+14**	134 (16)	131 (8)	-3	108 (23)	121 (12)	**+13**
**Lever arm dysfunction**											
Saraph et al.[14]	3.1	D	98 (22)	114 (16)	+16	128 (10)	118 (4)	-10	103 (20)	113 (18)	**+10**
	3.2	H	104 (24)	106 (10)	+2	132 (17)	128 (22)	-4	113 (18)	114 (20)	+1
Dobson et al.[15]	2.9								109 (15)	114 (18)	+5
Ounpuu et al. [16]	1.0		77 (17)	82 (14)	+5^x^	125 (30)	121 (30)	-4^x^	84 (29)	85 (25)	+1^x^
	5.0		77 (17)	102 (21)	+25^x^	125 (30)	116 (26)	-9^x^	84 (29)	102 (29)	+18^x^
**Multi-level tendon lengthening surgery**											
Steinwender et al.[18]	3.4		95 (18)	107 (13)	**+12**	140 (16)	132 (11)	**-8**	110 (26)	118 (13)	+8
Adolfsen et al.[19]	1.9		92 (11)	102 (14)	**+10**	136 (11)	128 (13)	**-8**	105 (16)	109 (17)	+4
Bernthal et al. [20]	1.7		66 (20)	75 (20)	**+9**	110 (32)	94 (32)	-16	63 (30)	69 (40)	+6
**Gait pattern**											
Cruz et al.[22]	1.5								83 (34)	84 (31)	+1
Dreher et al.[23]	1.0	RF	80 (20)	80 (20)	0	113 (28)	112 (29)	-1	80 (30)	80 (30)	0
	1.2	NRF	80 (20)	80 (20)	0	119 (18)	113 (25)	-6	80 (30)	80 (30)	0
Dreher et al.[24]	1.2	RF	80 (20)	80 (20)	0	125 (24)	115 (19)	**-10**	90 (30)	90 (20)	0
	1.0	PRF	80 (20)	80 (20)	0	110 (26)	82 (33)	**-28**	70 (20)	60 (30)	-10
	8.6	RF	80 (20)	100 (20)	**+20**	125 (24)	114 (21)	-9	90 (30)	100 (30)	+10
	8.9	PRF	80 (20)	80 (20)	0	110 (26)	96 (24)	-14	70 (20)	70 (30)	0
Presedo et al.[25]	2.2		109 (24)	116 (14)	-7	70 (30)	90 (20)	+20
**Operative technique**											
Dreher et al.[27]	1.0		90 (20)	80 (20)	-10	124 (23)	109 (31)	**-15**	90 (20)	80 (30)	**-10**
	3.3		90 (20)	90 (20)	0	124 (23)	117 (18)	-7	90 (20)	90 (20)	0
	8.6		90 (20)	100 (20)	**+10**	124 (23)	113 (17)	**-11**	90 (20)	100 (20)	**+10**
Metaxiotis et al.[29]	3.1					118 (25)	103 (24)	**-15**			

*Abbreviations*: cm, centimetres; min, minutes; sec, seconds; SD, standard deviation; Pre, pre-operative; Post, post-operative; HM, medial hamstrings lengthening; HL, combined medial and lateral hamstrings lengthening; D, diplegia; H, hemiplegia; RF, distal rectus femoris transfer; PRF, prophylactic distal rectus femoris transfer; NRF, no distal rectus femoris transfer. *Colour coding*: Green boxes indicate a significant improvement, red boxes indicate deterioration and non-highlighted boxes indicate no change in gait parameters. Significant difference if p < 0.05.

**Table 4:** Changes in sagittal plane of the pelvis and hip kinematic data after SEMLS. Data expressed mean (standard deviation) and mean change.

Article	Follow-up time(yrs)	Sub-group	Pelvic range of motion (°)			Mean pelvic tilt (°)			Hip range of motion (°)			Min. hip flex. in stance (°)			Max. hip flex.in swing (°)		
			*Norm values*: *1*° – *5*°			*Norm values*: *10*° – *14*°			*Norm values*: *47*° – *50*°			*Norm values*: *-14*° –*-8*°			*Norm values*: *36*° – *39*°		
			Pre	Post	Mean change	Pre	Post	Mean change	Pre	Post	Mean change	Pre	Post	Mean change	Pre	Post	Mean change
**General multi-level surgery**																	
Dreher et al.[8]	1.0	HM				17 (8)	20 (7)	+3									
		HL				16 (10)	22 (10)	+6									
	3.1	HM				17 (8)	17 (8)	0									
		HL				16 (10)	17 (10)	+1									
	8.1	HM				17 (8)	18 (7)	+1									
		HL				16 (10)	20 (10)	+4									
Rutz et al.[9]	1.8					9 (5)	12 (8)	+3									
Saraph et al.[10]	3.3		10 (3)	6 (3)	**-4**	18 (6)	21 (6)	**+3**	43 (10)	47 (9)	**+4**	4 (11)	2 (8)	-2	46 (10)	48 (8)	+2
Saraph et al.[11]	1.0								44 (11)	46 (10)	+2	5 (11)	2 (9)	-3	49 (10)	48 (9)	-1
	2.3								44 (11)	46 (8)	+2	5 (11)	2 (9)	-3	49 (10)	48 (9)	-1
	4.4								44 (11)	44 (8)	0	5 (11)	-1 (9)	**-6**	49 (10)	43 (10)	**-6**
Zwick et al.[12]	3.8		10 (2)	6 (2)	**-4**	20 (8)	22 (6)	+2				5 (13)	3 (9)	-2			
**Lever arm dysfunction**																	
Saraph et al.[14]	3.1	D	10 (3)	9 (3)	-1	17 (3)	17 (6)	0	26 (12)	43 (4)	**+17**	13 (11)	5 (9)	-8	39 (8)	48 (6)	**+9**
	3.2	H	10 (4)	9 (3)	-1	15 (8)	18 (8)	+3	37 (6)	37 (8)	0	5 (7)	2 (13)	-3	42 (3)	38 (6)	-4
Dobson et al.[15]	2.9								41 (9)	37 (12)	-4	5 (9)	5 (8)	0	46 (7)	42 (7)	-4
Ounpuu et al.[16]	1.0		10 (5)	7 (4)	-3												
	5.0		10 (5)	8 (5)	-2												
**Multi-level tendon lengthening surgery**																	
Adolfsen et al.[19]	1.9		9 (3)	8 (2)	**-1**	19 (6)	21 (6)	+2	48 (9)	46 (12)	-2	2 (7)	3 (9)	+1	50 (8)	48 (10)	-2
Bernthal et al.[20]	1.7					18 (10)	21 (9)	+3	38 (12)	40 (11)	-2	15 (13)	11 (11)	**-4**	52 (12)	50 (11)	-2
**Gait pattern**																	
Rodda et al.[21]	1.0					14 (12)	28 (9)	**+14**				17 (16)	16 (12)	-1			
	5.0					14 (12)	24 (9)	**+10**				17 (16)	14 (11)	-3			
**Operative technique**																	
Metaxiotis et al.[29]	3.1		8 (2)	7 (2)	**-1**							10 (13)	1 (8)	**-9**			
Dreher et al.[30]	1.3	CBM				15 (6)	19 (7)	+4				10 (15)	4 (10)	-6			
	1.2	MTL				14 (8)	21 (8)	**+7**				6 (11)	6 (14)	0			
	9.2	CBM				15 (6)	17 (8)	+2				10 (15)	6 (11)	-4			
	9.1	MTL				14 (8)	17 (7)	**+3**				6 (11)	7 (11)	+1			
Thomson et al.[31]	1.0	MI				18 (3)	19 (7)	+1				7 (12)	8 (13)	+1	50 (8)	48 (9)	-2
		NMI				16 (5)	19 (10)	+3				9 (14)	8 (17)	-1	53 (9)	50 (11)	-3

*Abbreviations*: SD, standard deviation; Pre, pre-operative; Post, post-operative; HM, medial hamstrings lengthening; HL, combined medial and lateral hamstrings lengthening; D, diplegia; H, hemiplegia; CBM, conversion of biarticular muscles; MTL, multi-tendon lengthening; MI, minimally invasive SEML techniques; NMI, no minimally invasive SEML techniques. *Colour coding*: Green boxes indicate a significant improvement, red boxes indicate deterioration and non-highlighted boxes indicate no change in gait parameters. Significant difference if p < 0.05.

**Table 5:** Changes in sagittal plane of the knee kinematic data after SEMLS. Data expressed mean (standard deviation) and mean change.

Article	Follow-up time(yrs)	sub-group	Knee ROM (°)			Knee flex initial contact (°)			Min. knee flex in stance (°)			Max. knee flexion in swing (°)			Timing of peak knee flexion (%)		
			*Norm values*: *58*° – *63*°			*Norm values*: *2*°*– 9*°			*Norm values*: *2*°*– 5*°			*Norm values*: *57*° – *66*°			*Norm values*: *71*%– *72*%		
			Pre	Post	Mean change	Pre	Post	Mean change	Pre	Post	Mean change	Pre	Post	Mean change	Pre	Post	Mean change
**General multi-level surgery in general**																	
Dreher et al.[8]	1.0	HM				37 (17)	16 (12)	**-21**	17 (20)	0 (14)	**-17**						
		HL				45 (14)	19 (8)	**-26**	35 (23)	7 (12)	**-28**						
	3.1	HM				37 (17)	22 (11)	**-15**	17 (20)	9 (12)	**-8**						
		HL				45 (14)	20 (12)	**-25**	35 (23)	12 (16)	-23						
	8.1	HM				37 (17)	23 (10)	**-14**	17 (20)	12 (12)	-5						
		HL				45 (14)	23 (10)	**-22**	35 (23)	12 (16)	**-23**						
Saraph et a.[10]	3.3		40 (14)	51 (10)	**+11**	30 (12)	24 (7)	**-6**	15 (15)	8 (8)	**-7**	56 (11)	59 (6)	+3			
Saraph et al.[11]	1.0		41 (17)	56 (12)	**+15**				19 (16)	7 (10)	**-12**	60 (12)	63 (10)	+3			
	2.3		41 (17)	52 (9)	**+11**				19 (16)	8 (7)	**-11**	60 (12)	59 (8)	-1			
	4.4		41 (17)	47 (10)	**+6**				19 (16)	14 (7)	**-5**	60 (12)	61 (9)	+1			
Zwick et al.[12]	3.8					32 (15)	24 (9)	**-8**	17 (16)	9 (7)	**-8**	59 (13)	59 (7)	0	78 (5)	77 (2)	-1
**Lever arm dysfunction**																	
Saraph et al.[14]	3.1	D	40 (20)	46 (7)	+6	35 (15)	20 (12)	**-15**	24 (21)	10 (13)	-14	63 (13)	56 (6)	**-7**			
	3.2	H	29 (15)	41 (15)	**+12**	30 (9)	20 (5)	**-10**	21 (2)	11 (7)	**-10**	50 (16)	53 (9)	+3			
Dobson et al.[15]	2.9		41 (16)	44 (11)	+ 3	25 (10)	19 (7)	**-6**	8 (16)	11 (6)	+3	49 (9)	55 (8)	**+6**	76 (4)	75 (4)	-1
Ounpuu et al.[16]	1.0					34 (11)	23 (11)	**-11**				61 (11)	59 (10)	+2	79 (8)	77 (8)	-2
	5.0					34 (11)	24 (14)	**-10**				61 (11)	53 (10)	**-8**	79 (8)	77 (5)	-2
**Multi-level tendon lengthening surgery**																	
Adolfsen et al.[19]	1.9		44 (16)	48 (16)	+4	31 (8)	21 (10)	**-10**				56 (10)	54 (10)	-2	79 (5)	74 (3)	**-5**
Bernthal et al.[20]	1.7					52 (14)	35 (15)	**-17**	37 (19)	20 (18)	**-17**	67 (15)	52 (14)	**-15**	84 (7)	84 (5)	0
**Gait pattern**																	
Rodda et al.[21]	1.0					52 (7)	25 (9)	**-27**	44 (9)	13 (9)	**-31**						
	5.0					52 (7)	26 (10)	**-26**	44 (9)	17 (11)	**-27**						
Cruz et al.[22]	1.5		37 (12)	39 (14)	+2	34 (12)	26 (10)	**-8**				53 (11)	52 (9)	-1	82 (5)	80 (5)	**-2**
Dreher et al.[23]	1.0	RF	34 (13)	45 (11)	**+11**	35 (14)	24 (9)	**-11**	22 (17)	12 (10)	**-10**	56 (11)	56 (7)	0	82 (6)	79 (5)	**-3**
	1.2	NRF	32 (16)	34 (13)	+2	37 (10)	27 (10)	**-10**	23 (17)	14 (14)	**-9**	55 (9)	48 (9)	**-7**	83 (6)	80 (7)	**-3**
Dreher et al.[24]	1.2	RF	35 (11)	49 (12)	**+14**	27 (9)	17 (9)	**-10**	10 (11)	2 (12)	**-8**	45 (6)	51 (9)	**+6**	80 (6)	77 (4)	**-3**
	1.0	PRF	29 (15)	45 (14)	**+16**	49 (17)	21 (10)	**-28**	38 (20)	7 (14)	**-31**	67 (12)	52 (12)	**-15**	82 (5)	81 (6)	-1
	8.6	RF	35 (11)	47 (11)	**+12**	27 (9)	18 (9)	**-9**	10 (11)	5 (9)	-5	45 (6)	52 (9)	**+7**	80 (6)	77 (4)	**-3**
	8.9	PRF	29 (15)	40 (13)	**+11**	49 (17)	27 (9)	**-22**	38 (20)	15 (13)	**-23**	67 (12)	54 (7)	**-13**	82 (5)	81 (5)	-1
Presedo et al.[25]	2.2		30 (13)	41 (12)	**+11**							43 (4)	53 (1)	**+10**	83 (7)	77 (5)	**-6**
**Operative technique**																	
Svehlík et al.[26]	1.0		43 (17)	52 (18)	**+9**	29 (11)	16 (8)	**-13**	15 (15)	5 (8)	**-10**						
	2.0		43 (17)	53 (12)	**+ 0**	29 (11)	19 (7)	**-10**	15 (15)	6 (8)	**-9**						
	5.0		43 (17)	51 (14)	**+8**	29 (11)	21 (7)	**-8**	15 (15)	8 (7)	-7						
	10.0		43 (17)	48 (13)	**+5**	29 (11)	17 (7)	**-12**	15 (15)	8 (10)	**-7**						
Saraph et al.[28]	2.2					27 (10)	19 (10)	**-8**	9 (14)	4 (10)	-5	60 (12)	58 (7)	-2			
Metaxiotis et al.[29]	3.1.		32 (15)	45 (14)	**+13**	41 (15)	19 (13)	**-22**	29(23)	6 (14)	**-23**	59 (13)	51 (8)	**-8**	81 (7)	81 (5)	0
Dreher et al.[30]	1.3	CBM	30 (13)	44 (11)	**+14**	41 (14)	16 (10)	**-25**	28 (20)	5 (14)	**-23**	58 (12)	49 (10)	**-9**			
	1.2	MTL	33 (13)	48 (12)	**+15**	41 (16)	23 (6)	**-18**	28 (21)	7 (13)	**-21**	61 (13)	56 (8)	**-5**			
	9.2	CBM	30 (13)	39 (12)	**+9**	41 (14)	24 (8)	**-17**	28 (20)	13 (11)	**-15**	58 (12)	52 (9)	**-6**			
Thomson et al.[31]	1.0	MI	37 (16)	43 (14)	**+6**	41 (12)	24 (12)	**-17**	22 (18)	14 (14)	**-8**	59 (13)	57 (11)	-2	82 (5)	78 (4)	**-4**
		NMI	31 (14)	38 (12)	**+7**	46 (18)	32 (13)	**-14**	35 (23)	21 (17)	**-14**	66 (14)	60 (8)	-6	83 (4)	80 (5)	**-3**

*Abbreviations*: SD, standard deviation; Pre, pre-operative; Post, post-operative; HM, medial hamstrings lengthening; HL, combined medial and lateral hamstrings lengthening; D, diplegia; H, hemiplegia; RF, distal rectus femoris transfer; PRF, prophylactic distal rectus femoris transfer; NRF, no distal rectus femoris transfer; CBM, conversion of biarticular muscles; MTL, multi-tendon lengthening; MI, minimally invasive SEML techniques; NMI, no minimally invasive SEML techniques. *Colour coding*: Green boxes indicate a significant improvement, red boxes indicate deterioration and non-highlighted boxes indicate no change in gait parameters. Significant difference if p < 0.05.

**Table 6:** Changes in sagittal plane ankle kinematic data after SEMLS. Data expressed mean (standard deviation) and mean change.

Article	Follow-up time (yrs)	sub-group	Dorsiflex. at initial contact (°)			Max. dorsiflex. in stance (°)			Max. dorsiflex. in swing (°)		
			*Norm values*: *-1*° – *5*°			*Norm values*: *10*° – *15*°			*Norm values*: *2*° – *10*°		
			Pre	Post	Mean change	Pre	Post	Mean change	Pre	Post	Mean change
**General multi-level surgery**											
Saraph et a.[10]	3.3		-4 (13)	1 (7)	**+5**				-1 (14)	4 (6)	**+5**
Saraph et al.[11]	1.0		-4 (12)	1 (6)	**+5**	6 (15)	11 (5)	+5	-2 (14)	7 (7)	**+9**
	2.3		-4 (12)	1 (6)	+5	6 (15)	12 (6)	+6	-2 (14)	5 (7)	**+7**
	4.4		-4 (12)	5 (7)	**+9**	6 (15)	16 (6)	**+10**	-2 (14)	9 (8)	**+11**
Zwick et al.[12]	3.8		-4 (13)	1 (6)	**+5**				-1(14)	5 (5)	**+6**
**Lever arm dysfunction**											
Saraph et al.[14]	3.1	D	-17 (14)	-2 (9)	**+15**				-16 (16)	3 (9)	**+19**
	3.2	H	-23 (19)	-3 (4)	+20				-23 (18)	-1 (6)	**+22**
Dobson et al.[15]	2.9		-17 (11)	-7 (8)	**+10**	-1 (13)	11 (7)	**+12**	-11 (11)	-2 (7)	**+9**
Ounpuu et al.[16]	1.0					6 (14)	16 (5)	**+10**			
	5.0					6 (14)	15 (7)	**+14**			
**Multi-level tendon lengthening surgery**											
Adolfsen et al.[19]	1.9		-5 (8)	-2 (7)	**+3**	7 (9)	12 (8)	**+5**	-3 (9)	3 (8)	**+6**
Bernthal et al.[20]	1.7		-3 (11)	2 (9)	+5	11 (16)	17 (8)	**+6**			
**Gait pattern**											
Rodda et al.[21]	1.0		12 (10)	3 (9)	**-7**	29 (9)	17 (8)	**-12**			
	5.0		12 (10)	0 (6)	**-7**	29 (9)	15 (6)	**-14**			
**Operative technique**											
Svehlík et al.[26]	1.0		-18(10)	-4 (5)	**+14**	-6 (14)	8 (5)	**+14**			
	2.0		-18(10)	-4 (6)	**+14**	-6 (14)	10 (6)	**+16**			
	5.0		-18(10)	-1 (7)	**+17**	-6 (14)	13 (5)	**+19**			
	10.0		-18 (10)	-6 (7)	**+12**	-6 (14)	9 (7)	**+15**			
Dreher et al.[27]	1.0		-7 (10)	-1 (5)	**+6**	4 (12)	11 (6)	**+7**	-5 (11)	3 (4)	**+8**
	3.3		-7 (10)	-2 (7)	**+5**	4 (12)	12 (7)	**+8**	-5 (11)	3 (7)	**+8**
	8.6		-7 (10)	-2 (6)	**+5**	4 (12)	12 (6)	**+8**	-5 (11)	3 (6)	**+8**
Saraph et al.[28]	2.2		-17 (12)	-2 (6)	**+15**				-16 (12)	2 6)	**+18**
Metaxiotis et al.[29]	3.1		-5 (21)	-2 (9)	+3	7 (22)	13 (8)	+5	-2 (20)	4 (6)	+6
Dreher et al.[30]	1.3	CBM				9 (18)	14 (8)	**+5**			
	1.2	MTL				8 (13)	12 (6)	**+4**			
	9.2	CBM				9 (18)	15 (6)	**+6**			
	9.1	MTL				8 (13)	12 (7)	**+5**			
Thomson et al.[31]	1.0	MI				1 (18)	15 (8)	**+14**	-9 (13)	5 8)	**+14**
		NMI				14 (13)	17 (7)	+3	2 (15)	9 8)	**+7**

*Abbreviations*: SD, standard deviation; Pre, pre-operative; Post, post-operative; D, diplegia; H, hemiplegia; CBM, conversion of bi-articular muscles; MTL, multi-tendon lengthening; MI, minimally invasive SEML techniques; NMI, no minimally invasive SEML techniques. *Colour coding*: Green boxes indicate a significant improvement and non-highlighted boxes indicate no change in gait parameters. Significant difference if p < 0.05.

**Table 7:** Changes in transverse plane kinematic data after SEMLS. Data expressed as average (standard deviation) and mean change.

Article	Follow-up time (yrs)	Sub-group	Mean pelvic rotation (°)			Mean hip rotation (°)			Mean foot progression (°)		
			*Norm values*: *-2*° – *5*°			*Norm values*: *-5*° – *4*°			*Norm values*: *-12*°*– -4*°		
			Pre	Post	Mean change	Pre	Post	Mean change	Pre	Post	Mean change
**General multi-level surgeries**											
Rutz et al.[9]	1.8		11 (6)	8 (4)	- 3	16 (10)	12 (5)	- 4	29 (24)	14 (7)	**-15**
**Lever arm dysfunction**											
Dreher et al.[13]	1.0		0 (8)	1 (6)	+1	17 (14)	-1 (11)	**-18**	17 (16)	-3 (10)	**-20**
	3.3		0 (8)	1 (7)	+1	17 (14)	1 (14)	**-16**	17 (16)	-1 (11)	**-18**
	8.6		0 (8)	1 (7)	+1	17 (14)	4 (13)	**-13**	17 (16)	3 (11)	**-14**
Saraph et al.[14]	3.1	D	-8 (6)	-6 (7)	+2	20 (6)	3 (3)	**-17**			
	3.2	H	-16 (4)	-7 (7)	**+9**	24 (16)	-2 (6)	**-26**			
Dobson et al.[15]	2.9		-14 (6)	-5 (6)	**+9**	23 (7)	-2 (11)	**-21**	11 (16)	-13 (15)	**-24**
Ounpuu et al.[16]	1.0		-5 (7)	-2 (6)	+3	20 (8)	2 (10)	**-18**	5 (17)	-11 (16)	**-16**
	5.0		-5 (7)	-2 (8)	+3	20 (8)	4 (14)	**-16**	5 (17)	-12 (14)	**-17**
Kay et al.[17]	1.5	FDO	-3 (6)	0 (6)	**+3**	11 (10)	0 (16)	**-11**	14 (16)	2 (22)	**-12**
		NFDO	-6 (6)	-3 (6)	**+3**	3 (19)	2 (14)	-1	-8 (19)	-13 (14)	**-5**
**Multi-level tendon lengthening surgery**											
Bernthal et al.[20]	1.7		1 (7)	0 (7)	-1				7 (14)	-2 (14)	**-9**
**Operative technique**											
Thompson et al.[31]	1.0	MI	16 (11)	8 (7)	**-8**	14 (12)	1 (8)	**-13**	14 (18)	-7 (15)	**-21**
		NMI	16 (14)	12 (16)	-4	12 (13)	2 (10)	**-10**	15 (14)	0 (15)	**-15**

*Abbreviations*: SD, standard deviation; Pre, pre-operative; Post, post-operative; D, diplegia; H, hemiplegia; FDO, femoral derotation osteotomy; NFDO, no femoral derotation osteotomy; MI, minimally invasive SEML techniques; NMI, no minimally invasive SEML techniques. *Colour coding*: Green boxes indicate a significant improvement and non-highlighted boxes indicate no change in gait parameters. Significant difference if p < 0.05.

### Gait analysis

Table 2 provides an overview of the 3DGA data collection protocols per article. All articles, except for the article of Steinwender et al. [18], described that the children were asked to walk at a self-selected speed. With regards to their footwear, ten articles (42%) reported that the children walked barefoot, while this was not reported in the other articles. The distance of the walkways ranged between 7 to 15 meters and generally 3 to 5 trials were used for data analyses.

In total 19 gait parameters were identified within the systematic review, namely three temporal-distance parameters (Table 3), thirteen sagittal plane parameters (Tables 4 and 5) as well as three transverse plane parameters (Table 7). Normalized temporal-distance parameters, frontal plane kinematic parameters and overall gait pattern score such as the Gait Deviation Index (GDI) [32] could unfortunately not be included due to a limited number of articles that had reported on these parameters.

### Temporal-distance parameters

Seventeen articles (32 studies) reported cadence, stride length and walking velocity before and after SEMLS intervention (Table 3). In addition, 4 articles also reported reference norm values [14,16,19,22], which ranged from 118–130 steps per minute (steps/min) for cadence, 111–134 centimetre (cm) for stride length and 119–138 centimetre per second (cm/sec) for walking velocity. After SEMLS intervention, 46% of the studies showed a significant change in stride length [8,10–12,14,18–20,24,29], while 39% of the studies showed a significant change in cadence [8,10,11,18,19,24,27,29]. This resulted in a change in the walking velocity in 31% of the studies [8,10–12,14,27].

### Kinematic sagittal plane parameters

Pelvic range of motion (ROM) and mean pelvic tilt were the most commonly used pelvic parameters as reported in 12 articles (24 studies) (Table 4). Four articles [10,16,19,31] reported reference norm values for pelvic ROM (1–5°) and mean pelvic tilt (10–14°). After the SEMLS intervention, a change in pelvic ROM was found in 50% of the studies [10,12,19,29], while 24% of the studies [10,21,30] reported a significant change in mean pelvic tilt.

Hip ROM, minimum hip flexion in stance and, maximum hip flexion in swing phase were reported in 11 articles (19 studies) (Table 4). Four studies [10,19,21,31] reported reference norm values for hip ROM (47–50°), minimum flexion in stance (-14 –-8°) and maximum flexion in swing (36–39°). Hip ROM significantly changed in 22% of the studies [10,14], while minimum hip flexion in stance changed in 16% of the studies [11,20,29] and maximum flexion in swing changed in 18% of the studies [11,14] after the SEMLS intervention.

Knee ROM, knee flexion at initial contact (IC), minimum knee flexion in stance and maximum knee flexion were reported in 19 articles (40 studies) (Table 5). Six articles reported [10,16,19,21,22,31], reported reference norm values for knee ROM (58–63°), knee flexion at IC (2–9°), minimum knee flexion in stance (2–5°) and maximum knee flexion in swing (57–66°), while peak knee flexion generally was seen at 71–72% of a step cycle. Knee ROM changes were found in 81% of the studies [10,11,14,23–26,29–31], while knee extension at IC changed in all studies (100%) [8,10,12,14–16,19–24,26,28–31] and in 80% of the studies during stance phase [8,10–12,14,20,21,23,24,26,29–31]. In addition, Peak knee flexion during the swing phase changed in 53% of the studies [14–16,20,23–25,29,30], while the timing of the peak knee flexion changes in 53% of the studies [19,22–25,31], after the SEMLS intervention

Ankle dorsiflexion at IC, maximum dorsiflexion in stance and maximum dorsiflexion in the swing phase were reported in 15 articles (29 studies) (Table 6). Five articles [10,16,19,21,31] reported reference norm values for dorsiflexion at IC (-1–5°), maximum dorsiflexion in stance (10–15°) and maximum dorsiflexion in the swing phase (2–10°). After the SEMLS intervention, 81% of the studies [10–12,14,15,19,21,26–28] reported a change in ankle dorsiflexion at IC, while 83% of the studies [11,15,16,19–21,26,27,30,31] reported an change in maximum dorsiflexion angle during stance. Maximum dorsiflexion angle during swing phase changed in 94% of the studies [10–12,14,15,19,27,28,31]

### Kinematic transverse plane parameters

Kinematics in the transverse plane, which included mean pelvic rotation, mean hip rotation and foot progression angles, were reported in 8 articles (14 studies) (Table 7). Four articles reported reference norm values for mean pelvic rotation (-2 − 5°), mean hip rotation (-5 − 4°) and foot progression (-12 − -4°). After the SEMLS intervention, internal and external rotation of the pelvis changed in 36% of the studies [14,15,17,31], while a change in internal and external rotation for the hip were found in 85% of the studies [13–17,31]. Foot progression changed significantly in all studies (100%) [9,13–17,20,31].

## Discussion

This is the first systematic review that provides an overview of the different SEMLS interventions within different SEMLS focus areas in children with spastic CP and, their effects on 3DGA gait parameters. For this 510 articles were screened of which 24 articles met the strict inclusion criteria for this systematic review (see section 2.2 and Fig 1). As some articles contained more than one follow-up study and/or population group, 50 different SEMLS studies were included for review (Table 2). The studies were based on ambulatory patients with CP (GMFCS level I-III), with a great emphasis on children with spastic diplegia (88% of the studies), and into a lesser extend hemiplegia (21%), quadriplegia (13%) or included all types (8%). In total 19 commonly used gait parameters were identified, specifically three temporal-distance parameters (Table 3), thirteen sagittal plane parameters (Tables 4, 5 and 6) and three transverse plane parameters (Table 7).

Improvements, defined as significant changes getting closer to the reference norm values, were reported for stride length (46% of the studies [10–12,14,18–20,24,27]), pelvic ROM (50% [10,12,19,29]), hip ROM (22% [10,14]), minimal hip flexion in stance (16% [11,20,29]), knee ROM (81% [10,11,14,23–26,29–31]), knee flexion at IC (100% [8,10,12,14–16,19–24,26,28–31]), minimal knee flexion in stance (80% [8,10–12,14,20,21,23,24,26,29–31]), timing of peak knee flexion (53% [14–16,20,23–25,29,30]), dorsiflexion at IC (81% [10–12,14,15,19,21,26–28]), maximum dorsiflexion in swing (94% [10–12,14,15,19,27,28,31]), mean pelvic rotation (36% [14,15,17,31]) and mean hip rotation (85% [13–17,31]).

Mixed results of the SEMLS interventions were found for cadence (18% improvements [10,11,18,19], 21% deterioration [24,27,29]), velocity (24% improvements [10–12,14,27], 7% deterioration [27])), maximum hip flexion in swing phase (9% improvements [11], 9% deterioration [14]), maximum knee flexion in swing phase (14% improvements [15,24,25], 39% deterioration [14,16,20,23,24,29,30]), maximum ankle dorsiflexion in stance (79% improvements [11,15,16,19,21,26,27,30], 4% deterioration [20]), and mean foot progression (92% improvement [9,13,15–17,20,31], 8% deterioration [17]). Deterioration was reported for mean pelvic tilt parameter in 24% of the studies [10,21,30].

The focus areas of the SEMLS interventions varied substantially between the 24 articles and ranged from a general focus [8–12] to more specific focus such as a lever-arm dysfunction [13–17], multi-tendon lengthening (MTL) [18–20], specific gait patterns [21–25], and different operative techniques focus [26–31]. The effect of these SEMLS interventions, within each focus area, on 3DGA parameters are discussed below.

### General SEMLS interventions

With the aim to increase joint mobility (ROM), gait posture and muscle control, general SEMLS interventions included frequently psoas recessions [10–12], medial hamstring lengthening [8–12] and rectus femoris transfer [8,10–12] interventions.

Follow-up studies showed good results with these techniques resulting in improved pelvic [10,12], hip [10], and knee [10,11] ROM. In addition Saraph et al. [11] also reported an improvement in hip extension in stance, although this finding has not been found by others. Improvement in knee extension in stance is more commonly reported, with positive results from one to eight years post-operatively [8,10–12]. In line with this, good improvements have also been reported for dorsiflexion at IC and in the stance and swing phase, up to three years post-operatively [10–12]. This type of general SEMLS intervention therefore mainly seem to improve gait kinematics at a knee and ankle level, which is likely to also result in a better a weight acceptance and foot clearance during a gait cycle. This is supported by the work of Austrian research group [10–12], who also reported significant improvements in stride lengths and walking velocity after this type of general SEMLS interventions.

### SEMLS to improve lever-arm dysfunction

The main aims of lever-arm dysfunction SEMLS interventions are to improve gait patterns through a more neutral pelvic and hip alignment and external foot progression. Since lever-arm dysfunction is related to torsional deformities [1], the main effects of this type of interventions can be expected in the transverse plane. As hemiplegic CP children have substantially more internal pelvic rotation on the affected side (pelvic retraction) [14,15] than children with spastic diplegia [13,14], better results of this lever-arm dysfunction SEMLS intervention are found in hemiplegic CP children. With regards to hip rotation and foot progression angles each lever-arm dysfunction SEMLS interventions study [13–17] showed significant post-operative improvements, except for one study [17]. In this study, where no FDO was performed, no improvement was found for these parameters. Although, one should not over-interpret this finding, this result suggests that the role of a FDO within a lever-arm dysfunction SEMLS intervention might be important.

In addition to the transverse plane, three of the five lever-arm dysfunction studies [14–16], also reported on changes in sagittal plane gait parameters. Significant improvements after the lever-arm dysfunction SEMLS interventions were found in kinematic data of the knee and ankle [14–16], while no changes were found in pelvic gait parameters [14–16] and only Saraph et al. [14] found improved hip mobility in children with spastic diplegia post-operatively after a lever-arm dysfunction SEMLS intervention.

### Multi-tendon lengthening interventions

Three research groups [18–20] studied the outcomes of SEMLS utilizing only multi-tendon lengthening (MTL) interventions. Adolfsen et al. [19] and Bernthal et al. [20] reported significant improvements in certain knee and ankle kinematics. Adolfsen et al. [19] reported significant improvements in the timing of peak knee flexion after rectus femoris transfers, while Bernthal et al. [20], who did not include this transfer as part of the MTL SEMLS, did not report this change. Interestingly both studies showed significant improvements in stride lengths, while no change in walking speed was found [19,20].

Bernthal et al. [20] and Steinwender et al. [18] also reported on changes in the transverse plane. Bernthal et al. [20] found significant improvements in foot progression, one year after MTL SEMLS interventions. As Steinwender et al. [18] used less common gait parameters within their study, these parameters were not included in Table 7. However it is interesting to mention that Steinwender et al. [18] reported significant improvements in mean hip transverse plane angles at different phases of the gait cycle (double support, single support, second double support and swing phase), while pelvic transverse plane parameters pre-operatively fell within ranges of reference norm values [14,17,31] and did not change after the MTL SEMLS intervention [18,20]

### SEMLS to improve gait patterns

Five studies [21–25] performed SEMLS with the specific objective to treat gait patterns in children with CP, such as crouch gait, stiff knee gait and jump knee gait.

The aim of Rodda et al.’s [21] study was to correct severe crouch gait with specific SEMLS interventions. Although significant improvements were found in the knee and ankle angles during the stance phase, no changes were found in excessive hip flexion angle after the SEMLS intervention. In addition, an increased mean anterior pelvic tilt angle was found one and five years post-operatively. This deterioration can possibly be explained by the relatively low amount of psoas procedures and high amount of hamstring procedures [21].

Stiff-knee gait, which is characterized by reduced knee ROM in the sagittal plane were specifically targeted with a SEMLS intervention by Cruz et al. [22] and Presedo et al. [25], which included rectus femoris recessions as part of the SEMLS. Cruz et al. [22] and Presedo et al. [25] both found improvements in the timing of the peak knee flexion, while Presedo et al. [25] also found improvements in knee ROM and peak knee flexion during swing after the SEMLS intervention.

Dreher et al. [23,24] studied the effects of a SEMLS intervention without and with rectus femoris or prophylactic rectus femoris transfer in children with CP to improve their gait. Dreher et al. found significant improvements in peak knee flexion in all three groups post operatively, while knee ROM and knee flexion also improved in the rectus femoris transfer groups and peak knee flexion deteriorated in the group without rectus femoris transfer [23, 24]. One to eight years post operatively the prophylactic rectus femoris transfer patients showed an improved knee ROM with no change in the timing of knee flexion and a deteriorated peak knee flexion during swing phase [23].

Adolfsen et al. [19] studied the effect of specific gait SEMLS interventions on children with an excessive crouch knee gait and jump knee gait. The SEMLS intervention included a rectus femoris transfer, a medial hamstring lengthening and calf muscle lengthening (85% aponeurotic Gastrocnemius lengthening and 15% tendon Achilles lengthening). Although no changes were found in knee ROM and peak knee flexion during swing, significant improvement were found in knee flexion at IC as well as the timing of peak knee flexion [19].

### SEMLS and operative techniques

Seven studies [26–31] focused on a specific operation technique as part of the SEMLS intervention, such as the Baumann procedure, conversion of bi-articular muscle groups and the use of minimal invasive techniques.

The studies by Svehlík et al. [26], Dreher et al. [27] and Saraph et al. [28] all focused on using the Baumann procedure as part of their SEMLS intervention. Short and long-term improvement in knee [26,28] and ankle [26–28] position at IC and dorsiflexion during stance and swing [26–28] were found after the intervention. These significant changes lead to better weight bearing and foot clearing characteristics, resulting in improvements in stride length and walking velocity [27].

Metaxiotis et al. [29] and Dreher et al. [30] performed SEMLS interventions which were focused on converting bi-articular muscle groups to mono-articular muscle groups. Three years post- operatively, Metaxiotis et al. [29] reported improved pelvic and knee ROM and hip and knee extensions, which resulted in a reduced crouch gait. In support of this method, Dreher et al. [30] reported similar findings but without significant improvements in hip extension.

Thompson et al. [31] studied the difference between conventional SEMLS techniques and minimally invasive SELMS techniques. The minimally invasive SEMLS technique used derotation osteotomies using closed corticotomy and fixation with titanium elastic nails and percutaneous lengthening of muscles where possible. Although operation time, blood loss and time to mobility were significantly less in the minimally invasive group, similar improvements in gait kinematics were found [31].

### Considerations and limitations

Although this systematic review provides a good overview of which gait changes can be expected after a certain type of SEMLS intervention in children with spastic CP, the data need to be interpreted within the available literature and its detail. This review is based on 24 articles with limited demographic information and varying heterogeneity within the study cohorts. This limitation did not allow stratifying for age or functional level of the subjects, what could have provided interesting information. In addition, the wide variety of surgical techniques and range in patient populations made it impossible to conduct a meta-analysis with drawing clear overall conclusions. Although this is admirable in the future, the differences in surgical preference and approach by different surgeons around the world might prevent this. It also needs to be mentioned that only one study could be classified as an OXFORD level II [23], while most studies were classified as an OXFORD level III, with only two studies [30, 31] wherein the comparative groups were matched with control factors.

Therefore, this systematic review should be seen as an overview paper providing a framework for clinical discussions and research, and a summary of results that can be used by clinicians to enhance the communication with parents when considering SEMLS in their child. However, we want to emphasize that the outcomes of the studies can’t be generalised. Each child with CP and his/her situation is different and the influence of a variety of confounding factors has to be kept in mind when interpreting research studies. For example, the psychological and social well-being of the child and their families, rehabilitation procedures offered and financial situations (difference in low-, middle-, and high-income countries) will influence the external validity of each study. Another consideration to take into account is that this systematic review is based on the change in gait parameters, but it is important to also look at other outcome measures and approach this holistically (e.g. what is the influence of SEMLS on quality of life)

With regards to the gait analyses itself, 3DGA is seen as the gold standard, however, the gait data should be interpreted within the reliability of the gait measurement itself, and the subjective interpretation of the data might slightly vary between the different experts [3,33,34]. There is also a lack of description of 3DGA data collection protocols, as well as variability within the studies (Table 2), which might influence the results of the studies. Future research should aim to reach a consensus on a general 3DGA model. The use of an overall gait pattern score, such as the Gait Deviation Index (GDI) [32], and normalisation of temporal-distance parameters should be encouraged. In addition, alternative clinical statistics, such as Cohen effect sizes [35] and magnitude based inferences [22], can potentially add additional values to these studies next to the traditional statistical methods.

### Conclusion

This is the first systematic review article which provides an overview of the effectiveness of SEMLS interventions based on different 3DGA parameters in children with spastic CP. SEMLS interventions generally resulted in good improvement in most gait parameters, with the biggest improvements seen for knee ROM, knee flexion at IC and minimal knee flexion in stance phase, ankle dorsiflexion at IC, maximum dorsiflexion in stance and in swing phase, hip rotation and foot progression angles. However, based on the main focus of the SEMLS intervention (e.g. lever-arm dysfunction, gait pattern, multi-tendon lengthening interventions) and the patient’s characteristics (e.g. age, CP diagnoses) changes in gait parameters might slightly vary. The current overview provides a framework for clinicians, researchers and parents, although individual factors and/or adaptations of SEMLS techniques need to be taken into account when interpreting the findings of this systematic review. In addition, future research should aim to have consensus on reporting 3DGA results, include outcome measures with a holistic approach and provide more specific information about the participants (psychological and social well-being), rehabilitation programs and costs involved.

## References

[1] Gage J, Schwartz MH. Consequences of brain injury on musculoskeletal development In: Gage J, Schwartz MH, Koop SE, Novacheck TF, editors. The identification and treatment of gait problems in cerebral palsy. London: Mac Keith Press; 2009 pp. 107–129.

[2] Bell KJ, Ounpuu S, DeLuca PA, Romness MJ. Natural progression of gait in children with cerebral palsy. 2002;22: 677–682. 12198474

[3] Narayanan UG. Management of children with ambulatory cerebral palsy: an evidence-based review. 2012;32 Suppl 2: S172–S181. pii: 01241398-201209001-00020. 10.1097/BPO.0b013e31825eb2a6 22890458

[4] McGinley JL, Dobson F, Ganeshalingam R, Shore BJ, Rutz E, Graham HK. Single-event multilevel surgery for children with cerebral palsy: a systematic review. 2012;54: 117–128. 10.1111/j.1469-8749.2011.04143.x 22111994

[5] Moher D, Liberati A, Tetzlaff J, Altman DG. Preferred reporting items for systematic reviews and meta-analyses: the PRISMA statement. 2009;62: 1006–1012. pii: S0895-4356(09)00179-6. 10.1016/j.jclinepi.2009.06.005 21603045 PMC3090117

[6] OCEBM Levels of Evidence Working Group The Oxford Levels of Evidence 2. 2013.

[7] Slim K, Nini E, Forestier D, Kwiatkowski F, Panis Y, Chipponi J. Methodological index for non-randomized studies (minors): development and validation of a new instrument. 2003;73: 712–716. pii: 2748. 12956787 10.1046/j.1445-2197.2003.02748.x

[8] Dreher T, Vegvari D, Wolf SI, Geisbusch A, Gantz S, Wenz W et al Development of knee function after hamstring lengthening as a part of multilevel surgery in children with spastic diplegia: a long-term outcome study. 2012;94: 121–130. 10.2106/JBJS.J.00890 22257998

[9] Rutz E, Baker R, Tirosh O, Brunner R. Are results after single-event multilevel surgery in cerebral palsy durable? 2013;471: 1028–1038. 10.1007/s11999-012-2766-9 23283676 PMC3563809

[10] Saraph V, Zwick EB, Zwick G, Steinwender C, Steinwender G, Linhart W. Multilevel surgery in spastic diplegia: evaluation by physical examination and gait analysis in 25 children. 2002;22: 150–157. 11856920

[11] Saraph V, Zwick EB, Auner C, Schneider F, Steinwender G, Linhart W. Gait improvement surgery in diplegic children: how long do the improvements last? 2005;25: 263–267. pii: 00004694-200505000-00001. 15832134 10.1097/01.bpo.0000151053.16615.86

[12] Zwick EB, Saraph V, Linhart WE, Steinwender G. Propulsive function during gait in diplegic children: evaluation after surgery for gait improvement. 2001;10: 226–233. 11497367

[13] Dreher T, Wolf SI, Heitzmann D, Swartman B, Schuster W, Gantz S et al Long-term outcome of femoral derotation osteotomy in children with spastic diplegia. 2012;36: 467–470. pii: S0966-6362(12)00166-X. 10.1016/j.gaitpost.2012.04.017 22766044

[14] Saraph V, Zwick EB, Zwick G, Dreier M, Steinwender G, Linhart W. Effect of derotation osteotomy of the femur on hip and pelvis rotations in hemiplegic and diplegic children. 2002;11: 159–166. 11943992 10.1097/00009957-200204000-00014

[15] Dobson F, Graham HK, Baker R, Morris ME. Multilevel orthopaedic surgery in group IV spastic hemiplegia. 2005;87: 548–555. pii: 87-B/4/548. 10.1302/0301-620X.87B4.15525 15795209

[16] Ounpuu S, DeLuca P, Davis R, Romness M. Long-term effects of femoral derotation osteotomies: an evaluation using three-dimensional gait analysis. 2002;22: 139–145. 11856918

[17] Kay RM, Rethlefsen S, Reed M, Do KP, Skaggs DL, Wren TA. Changes in pelvic rotation after soft tissue and bony surgery in ambulatory children with cerebral palsy. 2004;24: 278–282. pii: 00004694-200405000-00008. 15105723 10.1097/00004694-200405000-00008

[18] Steinwender G, Saraph V, Zwick EB, Uitz C, Linhart W. Assessment of hip rotation after gait improvement surgery in cerebral palsy. 2000;66: 259–264. 11033916

[19] Adolfsen SE, Ounpuu S, Bell KJ, DeLuca PA. Kinematic and kinetic outcomes after identical multilevel soft tissue surgery in children with cerebral palsy. 2007;27: 658–667. 10.1097/BPO.0b013e3180dca114 17717467

[20] Bernthal NM, Gamradt SC, Kay RM, Wren TA, Cuomo AV, Reid J et al Static and dynamic gait parameters before and after multilevel soft tissue surgery in ambulating children with cerebral palsy. 2010;30: 174–179. 10.1097/BPO.0b013e3181d04fb5 20179566

[21] Rodda JM, Graham HK, Nattrass GR, Galea MP, Baker R, Wolfe R. Correction of severe crouch gait in patients with spastic diplegia with use of multilevel orthopaedic surgery. 2006;88: 2653–2664. ppi: 88/12/2653. 10.2106/JBJS.E.00993 17142416

[22] Cruz AI, Ounpuu S, DeLuca PA. Distal rectus femoris intramuscular lengthening for the correction of stiff-knee gait in children with cerebral palsy. 2011;31: 541–547. pii: 01241398-201107000-00012. 10.1097/BPO.0b013e31821f818d 21654463

[23] Dreher T, Gotze M, Wolf SI, Hagmann S, Heitzmann D, Gantz S et al Distal rectus femoris transfer as part of multilevel surgery in children with spastic diplegia—a randomized clinical trial. 2012;36: 212–218. pii: S0966-6362(12)00068-9. 10.1016/j.gaitpost.2012.02.017 22425637

[24] Dreher T, Wolf SI, Maier M, Hagmann S, Vegvari D, Gantz S et al Long-term results after distal rectus femoris transfer as a part of multilevel surgery for the correction of stiff-knee gait in spastic diplegic cerebral palsy. 2012;94: e142–10. pii: 1361622. 10.2106/JBJS.K.01300 23032593

[25] Presedo A, Megrot F, Ilharreborde B, Mazda K, Pennecot GF. Rectus femoris distal tendon resection improves knee motion in patients with spastic diplegia. 2012;470: 1312–1319. 10.1007/s11999-011-2019-3 21842297 PMC3314761

[26] Svehlik M, Kraus T, Steinwender G, Zwick EB, Saraph V, Linhart WE. The Baumann procedure to correct equinus gait in children with diplegic cerebral palsy: long-term results. 2012;94: 1143–1147. pii: 94-B/8/1143. 10.1302/0301-620X.94B8.28447 22844059

[27] Dreher T, Buccoliero T, Wolf SI, Heitzmann D, Gantz S, Braatz F et al Long-term results after gastrocnemius-soleus intramuscular aponeurotic recession as a part of multilevel surgery in spastic diplegic cerebral palsy. 2012;94: 627–637. 10.2106/JBJS.K.00096 22488619

[28] Saraph V, Zwick EB, Uitz C, Linhart W, Steinwender G. The Baumann procedure for fixed contracture of the gastrosoleus in cerebral palsy. Evaluation of function of the ankle after multilevel surgery. 2000;82: 535–540. 10.1302/0301-620x.82b4.9850 10855877

[29] Metaxiotis D, Wolf S, Doederlein L. Conversion of biarticular to monoarticular muscles as a component of multilevel surgery in spastic diplegia. 2004;86: 102–109. 14765875

[30] Dreher T, Vegvari D, Wolf SL, Klotz M, Muller S, Metaxiotis D et al Long-term effects after conversion of biarticular to monoarticular muscles compared with musculotendinous lengthening in children with spastic diplegia. 2013;37: 430–435. pii: S0966-6362(12)00325-6. 10.1016/j.gaitpost.2012.08.020 23018029

[31] Thompson N, Stebbins J, Seniorou M, Wainwright AM, Newham DJ, Theologis TN. The use of minimally invasive techniques in multi-level surgery for children with cerebral palsy: preliminary results. 2010;92: 1442–1448. pii: 92-B/10/1442. 10.1302/0301-620X.92B10.24307 20884985

[32] Schwartz MH, Rozumalski A. The Gait Deviation Index: a new comprehensive index of gait pathology. 2008;28: 351–357. 10.1016/j.gaitpost.2008.05.001 18565753

[33] Chang FM, Rhodes JT, Flynn KM, Carollo JJ. The role of gait analysis in treating gait abnormalities in cerebral palsy. 2010;41: 489–506. 10.1016/j.ocl.2010.06.009 20868880

[34] Wren TA, Gorton GE III, Ounpuu S, Tucker CA. Efficacy of clinical gait analysis: A systematic review. 2011;34: 149–153. pii: S0966-6362(11)00151-2. 10.1016/j.gaitpost.2011.03.027 21646022

[35] Cohen J. Statistical Power Analysis for the Behavioral Sciences. New Jersey: Lawrence Erlbaum Associates; 1988

